# A high-grade undifferentiated prostate malignancy in a young male suggestive of male adnexal tumour of Wolffian origin – A rare case and diagnostic challenge

**DOI:** 10.1016/j.eucr.2026.103359

**Published:** 2026-01-27

**Authors:** Rowan Klein Nulend, Kathleen Lockhart, Kevin Tree, Linh NK. Tran, Bashar Matti, Yelise Foon, Ali Moghimi, Daniel Wong, Jordan Butler, Lawrence HC. Kim, Audrey Wang, Manish I. Patel

**Affiliations:** a Westmead Hospital, Sydney, NSW, 2145, Australia; b University of Sydney, Sydney, NSW, 2050, Australia

**Keywords:** Prostate cancer, Mesonephric tumour, Youth and adolescent cancer

## Abstract

We report a rare case of a high-grade, poorly differentiated prostatic malignancy in a 24-year-old, initially presenting with haematuria and urinary retention following trauma. Imaging and cystoscopy revealed a large prostate mass. Although a final histopathological diagnosis could not be definitively determined, expert review suggests this may resemble a male adnexal tumour of Wolffian origin, an extremely rare pathology. The patient underwent neoadjuvant chemotherapy, followed by radical cystoprostatectomy and adjuvant radiotherapy. He remains in remission 18 months post-treatment. This case underscores the importance of thorough investigation and multidisciplinary management of rare, aggressive prostatic tumours in young patients.

## Highlights

• Rare presentation – Prostatic malignancies in young adults are exceptionally uncommon. This case emphasizes the need to consider malignant pathology even in patients outside the typical demographic.
• Diagnostic challenges – Despite advanced imaging, immunohistochemistry, genetic testing, and expert histopathology review, a definitive tumour subtype was difficult to establish. This underlines the limitations of current diagnostic tools in rare tumour biology.
• Importance of early, multidisciplinary intervention – Prompt recognition of haematuria as a red-flag symptom, coupled with imaging, enabled early diagnosis. Aggressive multimodal therapy within a multidisciplinary framework resulted in 12-month remission.

## 1 Introduction

Prostate cancer is the most common non-cutaneous malignancy among men, typically affecting older individuals and very rarely encountered in adolescents or young adults. When prostatic tumours do occur in younger populations, they are often aggressive and present at an advanced stage.^1^ These cases are associated with poor prognoses and are rarely documented, especially in Indigenous populations.^1^ This report presents a unique case of an aggressive, poorly differentiated prostatic malignancy in a 24-year-old Aboriginal male, highlighting the challenges in diagnosis, management, and the importance of early multidisciplinary intervention.

## 2 Case presentation

**Fig. 1:**
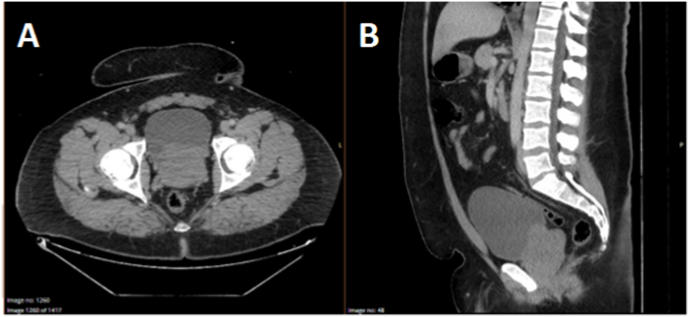
Panel A - Axial CT Imaging at Initial Presentation showing Irregular Prostatomegaly with Intravesical Protrusion Panel B - Sagittal CT Imaging at Initial Presentation showing Irregular Prostatomegaly with Intravesical Protrusion.

Our patient initially presented to Emergency with macroscopic haematuria and urinary retention immediately following a motor-vehicle accident. No traumatic injuries were identified on initial assessment. Multiphase CT imaging demonstrated prostatomegaly with intravesical protrusion (Fig. 1) but no overt discrete bladder masses or hydroureteronephrosis. He had no significant personal medical history or family history of malignancy, including prostate cancer.

An elective cystoscopy was performed to further investigate the patient's haematuria. This demonstrated abnormal solid, friable tissue contiguous with the prostate, extending to involve the bladder neck. There were no other discrete bladder masses, and the appearance of the urethra was otherwise normal. A transurethral resection was conducted, and histopathology revealed a poorly differentiated, high-grade malignancy of unclear histogenesis, invading muscularis propria of the bladder. Genetic and immunohistochemical testing were unable to determine a specific diagnosis, despite input from several tissue pathologists across multiple sites.

**Fig. 2:**
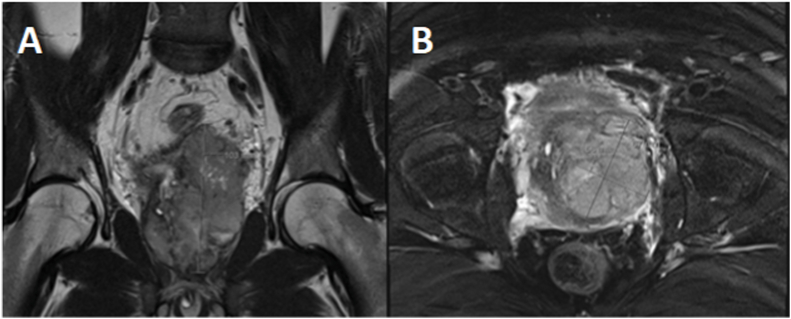
Panel A - Coronal MRI Imaging Following Transurethral Resection showing Irregular Prostatomegaly with Intravesical Protrusion and Internal Necrosis Panel B – Axial MRI Imaging Following Transurethral Resection showing Irregular Prostatomegaly with Intravesical Protrusion and Internal Necrosis.

**Fig. 3:**
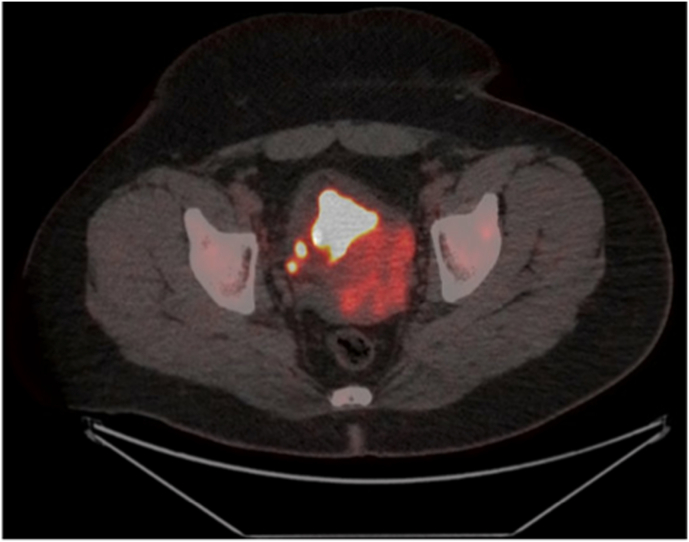
– Fused Axial FDG Positron Emission Tomography showing FDG Avidity within Prostate Gland suggestive of High Metabolic Activity.

His prostate specific antigen level was within normal limits at 1.75μg/L one month post transurethral resection. Pelvic MRI at this time revealed a residual 100x70 × 60mm heterogenous enhancing tumour with interspersed cystic spaces (probable necrosis), inseparable from the prostate gland at the base of the bladder (Fig. 2). The bladder appeared thickened and caudally contiguous with the tumour, though there was no overt invasion of surrounding structures. The seminal vesicles could not be identified. Positron emission tomography and further computed tomography staging scans revealed heterogenous intense FDG uptake (SUVmax 10.6) in the prostate mass, and FDG-avid (SUVmax 6.9) right para-rectal lymphadenopathy and internal and external iliac inguinal lymphadenopathy, without evident distant metastases (Fig. 3).

After extensive discussion at urological-oncology and sarcoma multidisciplinary meetings, the patient was referred for neoadjuvant chemotherapy (doxorubicin/ifosfamide/mesna). Neoadjuvant chemotherapy was given due to the provisional diagnosis of Ewing sarcoma (based on biopsy), which was excluded by eventual post-operative histopathology (following cystoprostatectomy). He showed good radiological response following two-and-a-half cycles of chemotherapy on serial MRI imaging, with tumour shrinking from 10x7x6cm to 7.5x3.6x3.5cm. Unfortunately the patient could not tolerate the side effects, hence discontinued neoadjuvant chemotherapy following two-and-a-half cycles.

**Fig. 4:**
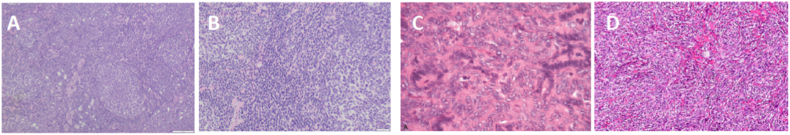
Panel A - Haematoxylin and Eosin Stained Slides showing a Prostate Tumour with Vaguely Nodular, Primitive Appearance Neoplasm with Areas of Spindling and Scattered Tubules Panel B – Haematoxylin and Eosin Stained Slides showing a Prostate Tumour with a Vaguely Nodular, Primitive Appearance Neoplasm with Areas of Spindling and Scattered Tubules Panel C – Haematoxylin and Eosin-Stained Slide of Different Patient's Male ATWO, showing Diffuse Solid Pattern and Focal Tubular Differentiation, as seen in our Patient^3^ Panel D – Haematoxylin and Eosin-Stained Slide of Female ATWO, showing Solid Growth Pattern with Spindle Cells and Small Nucleoli, as seen in our Patient^2^.

He then proceeded to radical cystoprostatectomy and ileal conduit formation. Histopathology confirmed a high-grade neoplasm, involving the majority of the prostate, left seminal vesicle, and left posterolateral bladder (including invasion of perivesical fibroadipose tissue), with negative, surgical margins. Lymph node dissection yielded 10 of lymph nodes, which were all negative for malignancy. Haematoxylin and eosin-staining showed a biphasic, vaguely nodular, primitive-appearing neoplasm with areas of spindling and scattered tubules (Fig. 4), similar to other reported cases of ATWO.^2^^,^^3^ Immunohistochemistry was negative for CK20, NKX3.1, GATA3, NKX2.2, among others, which in combination with histopathological appearance aided in excluding differentials such as urothelial or prostate carcinoma, and Ewing sarcoma. Further details surrounding immunohistochemical testing and relevant differentials are listed in Table 1.

**Table 1:** – Immunohistochemical testing results.

Immunohistochemical Test	Origin/Significance	Result
Cytokeratin AE1/AE3	Epithelial	Positive
CD56	NK cell, Haematopoietic, Neuroendocrine	Positive
Vimentin	Chromophobe RCC	Positive
P53	High grade neoplasm	Positive
CD99	Only when strongly positive: Ewing, Leukemia, Lymphoma	Patchy positivity
Cyclin D1	Lymphoma, Leukemia, Renal, Prostate Adenocarcinoma	Patchy positivity
GFAP	CNS	Patchy positivity
Glypican 3	Liver, Germ cell	Patchy positivity
BCL2	Lymphoma	Patchy positivity
P40	SCC	Focal
Chromogranin	Neuroendocrine	Negative
Synaptophysin	Neuroendocrine	Negative
GATA3	Urothelial, Breast	Negative
TTF-1	Thyroid, Lung	Negative
CK20	Urothelial, Merkel cell, Colon	Negative
NKX3.1	Prostate	Negative
NKX2.2	Ewing sarcoma	Negative
CD34	Haematopoietic	Negative
CDX2	Gastrointestinal	Negative
OCT4	Germ cell	Negative
CD117	GIST	Negative
Calretinin	Sex cord stromal, Mesothelial, adrenal, Mesonephric, FATWO	Negative
NUTM1	Germ cell	Negative
SSX	Synovial sarcoma	Negative
Desmin	Myogenic	Negative
DOG1	GIST	Negative
CD3	T cell	Negative
LCA	Haematopoietic	Negative
SOX 10 (A8,A2,A1).	Melanoma	Negative
WT1	Mesothelioma	Negative
Myogenin/Myoglobin	Rhabdomyosarcoma	Negative
P63	Basal cells	Negative
SALL4	Germ cell	Negative
Pan TRK	NTRK Fusions	Negative
BRAF (A2, A4)	Melanoma, Colorectal	Negative
INI1	Epithelioid sarcoma (if lost)	Retained

Again, following referral to multiple subspecialised tissue pathologists, the final histopathological diagnosis remained elusive, remaining that of a poorly differentiated, high-grade malignancy. Although multiple differentials were suggested, the tissue was determined to most-closely resemble a male adnexal tumour of Wolffian origin, an exceptionally rare diagnosis with only nine previous case reports. This is based on the glands and tubules scattered amongst solid sheets of round, fusiform and spindle cells expressing keratins, CK7, CD10, PAX2, PAX8 and AR, but negative for WT1 and SF1 (Table 1), and is further supported by the presence of mesonephric remnants immediately adjacent to the tumour. Molecular cytogenetics performed revealed no gene fusions or somatic variants (RNASeq-CHW Paediatric Solid Tumour v3.0 panel {59 genes}).

The patient then completed 21 42Gy fractions of adjuvant radiotherapy to the prostate bed and nodes and remains in remission on CT surveillance 18 months following cystoprostatectomy.

## 3 Discussion

Prostate cancer is the most commonly diagnosed non-cutaneous malignancy in males.^4^ It is estimated to affect one in six males by the age of 85 years in developed countries, with adenocarcinoma being most common subtype by far. However, it is an extremely rare tumour in young adults.

Case reports have described primary prostatic malignancy in young adults and adolescents, including rhabdomyosarcoma (most frequently in this group),^5^^,^^6^ high grade adenocarcinoma, leiomyosarcoma and other subtypes such as stromal or Ewing sarcomas 7, 8, 9. Typically, these tumours are highly aggressive, tend to be more advanced at time of diagnosis, and have a poor prognosis.

Beyler et al. conducted a U.S National Cancer institute database review of prostate carcinoma cases in the United States from 2000 to 2015. The youngest patients among this cohort were thirteen and sixteen years old.^1^ Most common presenting symptoms included lower urinary tract symptoms (LUTS) - characteristically dysuria, haematuria, urinary frequency, or pelvic pain.

Unlike these cases described in the literature, our patient had not experienced any LUTS prior to presentation. Instead, a suspicious lesion was detected on imaging following a motor-vehicle accident, which coincided with his first episode of haematuria and acute urinary retention. Given that no other injuries were identified, it is difficult to ascertain whether this haematuria triggered by traumatic injury, or coincidentally around the same time as the patient's motor-vehicle accident. However, it is likely that imaging was expedited due to the preceding trauma. This allowed for timely endoscopic investigation and management.

Female adnexal tumour of Wolffian origin (ATWO) is a rare neoplasm in women, derived from mesonephric (Wolffian) remnants, with around 100 reports in female patients and approximately 10 % of these demonstrating malignant potential.^10^ While the mesonephric ducts regress in females, in males they form reproductive organs including the epididymis, seminal vesicles and vas deferens. Extremely rare reports describe tumours in male patients which resemble ATWO, including reports in the seminal vesicles, para-urethral (at prostate apex),^3^^,^^11^ ischiorectal fossa,^12^ para- and intra-testicular,^13^^,^^14^ with a total of nine reported cases in literature. The majority of these cases were detected incidentally or during investigation for LUTS. Although the median reported age for male ATWO is 48 years, the youngest report was in a 29 year old man, with a tumour in a seminal vesicle.^15^ Histologically, our case shows similar immunohistochemical and pathological features to these reported cases of both ATWO in both males and females. This interpretation is based on the glands and tubules being scattered among sheets of round, fusiform and spindle cells (expressing keratins CK7, CD10, PAX2 and PAX8), and is further supported by the presence of mesonephric remnants immediately adjacent to the tumour. Furthermore our case shares a similar clinical course of a relatively indolent onset without evidence of distant metastasis. Although no specific treatment guidelines exist, the mainstay in treatment throughout literature has been via radical surgical excision, as was performed for our patient. Chemo- and radiotherapies have been used, however their exact therapeutic benefit remains unclear in this cohort.^10^

Although significant difficulty was encountered during histopathological review, this tumour is thought to closely resemble an adnexal tumour of Wolffian origin, an exceptionally rare phenomenon, especially in male patients.

## 4 Conclusion

This case highlights the importance of complete diagnostic workup of red-flag symptoms and signs such as macroscopic haematuria, including both imaging and endoscopic assessment, even in patients who are considered low-risk based on demographic factors. Prostatic tumours in young patients are exceedingly rare but have a poor prognosis. These tumours should be managed in a multidisciplinary setting, with early and aggressive management. To the best of our knowledge, this is the first case of this nature reported in the prostate, in this age group. This adds to the existing literature surrounding both male ATWO and prostate tumours in young males.

## CRediT authorship contribution statement

**Rowan Klein Nulend:** Writing – original draft, Visualization, Validation, Project administration, Methodology, Investigation, Formal analysis, Data curation, Conceptualization. **Kathleen Lockhart:** Writing – review & editing, Visualization, Validation, Project administration, Conceptualization. **Kevin Tree:** Writing – review & editing, Methodology, Data curation. **Linh NK. Tran:** Writing – review & editing, Visualization, Resources. **Bashar Matti:** Writing – review & editing, Resources, Investigation. **Yelise Foon:** Resources, Investigation, Formal analysis, Data curation. **Ali Moghimi:** Writing – review & editing, Resources, Investigation. **Daniel Wong:** Writing – review & editing, Resources, Investigation, Formal analysis. **Jordan Butler:** Writing – review & editing, Validation, Resources, Methodology, Formal analysis, Data curation. **Lawrence HC. Kim:** Writing – review & editing, Visualization, Supervision, Project administration. **Audrey Wang:** Writing – review & editing, Supervision, Project administration, Investigation. **Manish I. Patel:** Writing – review & editing, Supervision, Resources, Data curation, Conceptualization.

## Funding

This research did not receive any specific grant from funding agencies in the public, commercial, or not-for-profit sectors.
